# Dilemmas in recovery-oriented practice to support people with co-occurring mental health and substance use disorders: a qualitative study of staff experiences in Norway

**DOI:** 10.1186/s13033-018-0211-5

**Published:** 2018-06-07

**Authors:** Eva Brekke, Lars Lien, Kari Nysveen, Stian Biong

**Affiliations:** 1 Norwegian National Advisory Unit on Concurrent Substance Abuse and Mental Health Disorders, Innlandet Hospital Trust, Post box 104, 2381 Brumunddal, Norway; 2 Faculty of Health and Social Sciences, University of Southeast Norway, Post box 235, 3603 Kongsberg, Norway; 3 Faculty of Social and Health Sciences, Inland Norway University of Applied Sciences, Post box 400, 2418 Elverum, Norway

**Keywords:** Recovery-orientation, Co-occurring disorders, Mental health service provision, Staff perspective, Qualitative methods

## Abstract

**Background:**

Recovery-oriented practice is recommended in services for people with co-occurring mental health and substance use disorders. Understanding practitioners’ perceptions of recovery-oriented services may be a key component of implementing recovery principles in day-to-day practice. This study explores and describes staff experiences with dilemmas in recovery-oriented practice to support people with co-occurring disorders.

**Methods:**

Three focus group interviews were carried out over the course of 2 years with practitioners in a Norwegian community mental health and addictions team that was committed to developing recovery-oriented services. Thematic analysis was applied to yield descriptions of staff experiences with dilemmas in recovery-oriented practice.

**Results:**

Three dilemmas were described: (1) balancing mastery and helplessness, (2) balancing directiveness and a non-judgmental attitude, and (3) balancing total abstinence and the acceptance of substance use.

**Conclusions:**

Innovative approaches to practice development that address the inherent dilemmas in recovery-oriented practice to support people with co-occurring disorders are called for.

**Electronic supplementary material:**

The online version of this article (10.1186/s13033-018-0211-5) contains supplementary material, which is available to authorized users.

## Background

Recovery-orientation is increasingly recommended in practice guidelines for community mental health and addiction services across countries [1]. An understanding of recovery as a personal and social process that surpasses symptom reduction is increasingly accepted in the fields of mental health and substance use [2–4]. The individual is considered the central actor and decision maker in his or her recovery, each person’s unique experiences are considered important, structural factors are recognised, and everyday life is acknowledged as a central arena for change [5]. While recovery may occur regardless of professional help [6], relationships with professional helpers often play an important role in the recovery process of persons with co-occurring mental health and substance use disorders (co-occurring disorders) [7], but may constitute both barriers and facilitators [8]. Underpinning the recovery movement is the intention to make services available and beneficial from the perspective of service users and to promote citizenship and civil rights.

Recovery-oriented practices have been defined in different ways across countries and services. One such definition is that they “*identify and incorporate a person’s own goals, interests, and strengths in the effort to support the person’s own efforts to manage his or her condition while pursuing a meaningful life in the community”* [9]. A qualitative analysis of recovery-oriented practice guidelines from several countries conceptualised recovery-oriented practice into four domains: promoting citizenship, organisational commitment, supporting personally defined recovery, and working relationship [10]. Norwegian health authorities recommend that *“the person’s own resources should be supported throughout treatment in a way that leads to an improved quality of life”* [11].

Concern has been raised regarding the potential misuse of ‘recovery’ in the transition from a concept developed by people with lived experience into a concept defined by staff, researchers, and service developers [12, 13]. Previous research suggests that staff perceptions of recovery-oriented practice may differ from those of service users, that recovery-oriented practice may be combined with seemingly incompatible practices, such as formal and informal coercion [14, 15], and that it may pose dilemmas to practitioners [16]. While recovery has been suggested as an organising principle for integrating mental health and addiction services [17], different definitions of recovery in mental health services and substance use services may pose challenges for practitioners addressing both issues [18, 19]. Also, staff may experience competing priorities between recovery principles and structural demands, such as financial resources and time [20].

In spite of an increasing knowledge base for the recommendation of recovery-oriented practice, a gap seems to exist between recommendations and actual practice, a main challenge being the lack of a shared understanding of what recovery-oriented practice means, based on multi-stakeholder views [20]. Exploring and describing challenges, paradoxes and dilemmas faced by practitioners in recovery-oriented services within different contexts may be a key component in the process of implementing recovery principles in day-to-day practice [10].

The aim of this study is to explore and describe staff experiences of dilemmas in recovery-oriented community practice to support people with co-occurring disorders in a Norwegian context. ‘Dilemma’ is understood as a situation in which a difficult choice has to be made between two or more alternatives, especially ones that are equally undesirable.

## Methods

### Context

This study is part of a larger project to investigate recovery-orientation in services in a Norwegian local authority area, containing agricultural areas, forested areas, and two community centres (< 6500 inhabitants). The project, which has an exploratory and descriptive purpose, has included individual interviews with residents with co-occurring disorders exploring what recovery means [21] and how professional helpers may contribute to recovery [7]. Interviews with family members have been conducted and results will be sought published. Results from these studies have been communicated to practitioners and leaders in the services, who were committed to developing recovery oriented services. Researches have otherwise not directed the local practice development.

Norwegian primary health care is run by local authorities, whereas hospital trusts are responsible for secondary and tertiary care. Municipal and specialised services share responsibility for providing services to people with co-occurring disorders. Since 2012, national guidelines have recommended recovery-oriented practice in Norwegian health and social services for people with co-occurring disorders [11]. Recovery-orientation was defined by services as recruiting peer support workers as part of staff at different system levels, and explicitly basing interventions on what was important for the person seeking help rather than their psychiatric diagnosis. As a tool for this, feedback informed treatment (FIT) was implemented in the services, which is a method for systematically getting feedback from the client on how the alliance and progress of treatment is experienced, and adjust interventions according to this [22].

Drawing on literature on collaborative research [23] and user involvement in research [24], a group of six people from the local community advised the authors throughout the process. Aiming to include different groups affected by the study [25], these were two people with lived experience of co-occurring disorders, one family member of a person with co-occurring disorders, one practitioner, the leader of the local peer support centre, and one experienced practitioner, who is the third author of this article. The group has had an advisory function throughout the process from planning the study through developing the interview guide, deciding the recruitment strategy and understanding the results in a local context.

### Data collection

Based on their specific experiences with recovery-oriented practice to support people with co-occurring disorders, which were considered relevant in answering the study aim [26], all members of the local mental health and addiction team were invited to participate in the study. The leader of the services communicated the invitation, which all team members accepted. Participants were community support workers (2), mental health workers (2), peer support workers (2), specialist nurses (2), social workers (1), and psychologists (1).

Three focus group interviews [27] were conducted over 2 years. Six to eight team members were present at each interview. The first interview lasted 90 min, and the second and third lasted 60 min. All interviews were led by the first and third authors. In order to facilitate participation, the interviews were carried out at the time of the weekly team meeting, in a meeting room at the team’s office building. In the first interview, participants were asked to describe their current practice in the field of co-occurring disorders (see Additional file 1). In the second and third interviews, participants were asked to describe their experiences with recovery-oriented practice with this group of citizens (see Additional file 2).

Between the first and second interview, seven 1-h meetings with team members were arranged over the course of 6 months, aiming to encourage reflection on their own practice in relation to recovery principles. The third and first author participated in all of these meetings and the fourth author participated in one of the meetings.

### Reflexive comments

The first and third authors, a clinical psychologist and a specialist nurse, knew some of the participants as collaborative partners from former jobs. The second and fourth authors, who are both professors with background from mental health and substance use treatment as psychiatrist and nurse, respectively, did not know the team members from before. All authors support a humanistic, person-centred approach to mental health and substance use treatment, guiding a common interest in recovery-orientation. Within this, the authors have adopted an intentionally non-judgmental and non-directive attitude in exploring recovery-oriented practice in this particular context, which has also been communicated to participants.

### Data analysis

Interviews were tape recorded and transcribed verbatim by the first author. Before the second and third interviews, transcripts from the previous interviews were read through in order to prepare and plan the interview situation. A full analysis was only carried out after all three interviews had been transcribed. Here, thematic analysis [28] was used. An explicit aim of the analysis was to provide a detailed and nuanced account of *dilemmas* in the participants’ descriptions. Within this aim, the analysis was inductive. An attempt was made to bracket the researcher’s pre-understanding during analysis, although complete bracketing is acknowledged to be impossible [29]. Themes were identified on a semantic level, based on the surface meaning of participants’ descriptions, with analysis moving from descriptions to interpretation. Firstly, transcripts were read several times while notes were taken, to enable familiarisation with the data. Secondly, the data set was read through systematically, giving equal attention to each data item, and content was coded by tagging and naming selections of text using the computer software QSR NVivo 10. When all data had been coded and collated, codes were sorted into potential themes. All collated extracts for each potential theme were then read through, and themes were adjusted based on the criteria of internal homogeneity and external heterogeneity. Following this process, the entire data set was read through, considering the validity of the candidate themes in relation to the interviews, including coding of additional data that had been missed during the first coding process. A detailed analysis was carried out for each theme; here, data extracts were organised into coherent accounts with accompanying narratives, and the essence of each theme was identified. This led to the creation of an analytic narrative, which constitutes “Results” section of this paper.

### Ethics

The study was approved by the Norwegian Centre for Research Data (Case No. 42244). Informed consent was a requirement for participation. The fact that team members were recruited by their leader may raise the question of whether participation was indeed voluntary, even though informed consent was a prerequisite for participation. The leader was not present during interviews and all information has been anonymised in order to ensure the confidentiality of participants.

## Results

Three dilemmas were described by practitioners regarding recovery-oriented practice with people who have co-occurring disorders: (1) balancing mastery and helplessness, (2) balancing directiveness and a non-judgmental attitude, and (3) balancing total abstinence and the acceptance of substance use.

### Balancing mastery and helplessness

In their daily practice with people with co-occurring disorders, team members described a challenge in determining how much help they should offer and how much responsibility they should put on the service user. They described that intervening with too much practical help might lead to disempowerment of the person and dependence on services, while not intervening might leave people in a deadlock situation which also hinders change.

*“Well, it’s about cooperating, playing as a team, advising them. There’s a fine line here in that we’re not supposed to take over their tasks, but at the same time you have to stand behind them a bit and be a motivator and push them. But you also have to try and give them the feeling that they can master things by themselves. That you don’t do everything for them. It’s not good to make people helpless.” (Interview 1)*

The team described that some service users had life challenges which made it unreasonable to expect them to assume responsibility for making changes, for instance discrimination in the housing market, a lack of social network, poverty, unfair treatment from health and social services, lack of everyday coping skills, cognitive challenges, and a bad reputation in the local community. Practicing living skills, such as taking out the garbage, and making arrangements to secure the housing and economy, such as a standing order for paying the rent, were described as interventions to meet these challenges. The team described acting like *an extended arm* into the system to ensure equal access, and acting like *a buffer* in the face of unfair treatment.

*“Another thing I’ve seen about people with substance use and mental health problems is that they’re often not respected when they come to an office on their own. They’re not listened to. (…) So we often come along as an extended arm.” (Interview 1)*

Sometimes, team members helped service users more than usual in order to enhance their motivation for change, even if the outcome was uncertain.

*“Now and then you start out on one of those journeys that are kind of chaos projects. I’m in one of those now, where I kind of do lots of things that I feel I shouldn’t be doing. But I’ll continue as far as I have decided, to see if it can stabilise things enough for the person to either go back to how things were, and be left alone, or maybe be motivated to take a different path. Because there are some things you just have to do, and you think that it’s worth it, but… I don’t know how it will end, you know, and whether the person will be defined as outside the services, a dropout, who won’t get any help… I don’t know.” (Interview 3)*

Scarce resources made it necessary to prioritise between tasks, and avoid doing tasks that were not within their responsibility. Working towards agency and internal motivation for change was seen as more effective than helping out with practical things.

*“If you want a change, you need to do something different. Holding down a job, that’s not easy, it’s based on your own efforts. You can’t come in here every one or 2* *weeks and expect me to make a change in you. Here you are, just like that. It needs to come from within. (…) So it’s like, how do you work with change? How do you make people understand change, and become active?” (Interview 3)*

Another dilemma concerned how much effort to invest when people did not attend. The team described that reaching out may enable trust and strengthen the alliance, but also means less time spent with those who comply. Making people responsible for attending was considered as potentially empowering. The team described a change from more outreach in the first interview to less outreach at the time of the last interview.

*“I think that I spend 70*–*80 percent of my time in the field and 20*–*30 percent inn the office. If it is a burden to them to attend an appointment, of course I’ll meet them out of the office.” (Interview 1)*

*“If it’s hard to get in touch with a service user and they’re not interested, then I say, ‘OK, well then you can come back when you’re a bit more interested’. And I don’t run after them like I used to do. Before, people did a lot to try to get hold of them, but I actually don’t do that anymore. (…) And when I don’t run after them, they have to kind of take some of the responsibility themselves, they have to contact us if they want help.” (Interview 3)*

Participants suggested that a solution to this dilemma may be to define and limit one’s areas of responsibility. One example of this was to state that they worked primarily with addressing substance use, and that those who were not interested in this should rather be followed up by other services. Another solution may be to offer extensive practical help in chaotic or crisis situations, while gradually transferring more responsibility to the service user. The team also had a policy where it was easy to re-establish contact without waiting time.

### Balancing directiveness and a non-judgmental attitude

A central aspect of recovery-oriented practice is that treatment goals should be based on what is important for the person seeking help. Practitioners described two possible pitfalls in connection with this principle in their day-to-day practice. On the one hand, judging the way people live their lives was described as paternalistic and ethically problematic. On the other hand, an “anything goes” approach might well lead to indifference.

Team members reported that adopting a non-judgemental attitude was an essential principle in their day-to-day practice.

*“We shouldn’t judge, we should listen to the person who’s actually living that life. And we’re all different, so we need to respect each individual, based on how he experiences his life and when he thinks his life is all right.” (Interview 2)*

However, when service users were content with living conditions that team members saw as unsatisfactory or undignified, respecting the other’s view was described as difficult.

*“In this FIT stuff, the service user’s supposed to say how he feels. But then the question is: How he feels, in relation to what? What’s your reference frame for feeling OK? For someone who’s always been on drugs, and lives in a crappy little bedsitter and has practically nothing, eats once a day, but he says he feels OK! But in my world, he’s not OK. (…) Should we be working to keep it like this, or what?” (Interview 2)*

The team suggested that people with co-occurring disorders may have internalised a belief that they deserve little, due to past experiences with oppression and scarcity. Hence, directly accepting the service user’s point of view may reinforce hopelessness and low expectations.

*“The starting point is that we ask everyone: ‘What’s important to you?’ At the same time, we can kind of dare to have slightly higher goals. Maybe particularly us, who work with substance abuse, we can dare to say: ‘You know what, we believe you can achieve a lot’. Or, you know, we strongly believe they can get better, even in areas where they maybe don’t believe it themselves.” (Interview 2)*

Team members described that one way to manage this dilemma was to introduce one’s own ideas in ways that make people feel that they have figured them out themselves.

*“You need to keep planting little seeds, which gradually give the person knowledge and ideas, so that people may start changing the way they think and maybe sort things out. Even though we’re the ones who’ve figured it out for them, the process actually makes them feel that they’re the ones who’ve sorted it out. But we plant some seeds, and when they start to blossom, that’s when it gets really interesting.” (Interview 2)*

### Balancing total abstinence and the acceptance of substance use

A third dilemma described by practitioners concerned relating to substance use in a recovery-oriented way.

Team members described that a professional, non-moralistic attitude towards substance use, including support and hopefulness in the face of relapse, enabled trust and honesty in the relationship with service users.

*“It’s important to establish a relationship when we’re out there, so that the service users who may not be too optimistic don’t feel that we’re moralisers, that we kind of tell them: ‘Oh dear, you’ve been taking drugs, haven’t you’. You must be there for them and try your best.” (Interview 1)*

However, accepting substance use was seen as a sign of giving up, denying people the opportunity to change. On the one hand, team members acknowledged the potential of change regardless of substance use. On the other hand, they feared that support without addressing substance use may enable the latter.

*“I think we’ve been too good at tidying up in the consequences of substance use. (…) Because if we keep tidying up when a crisis comes, it’ll be quite nice to just carry on taking drugs.” (Interview 3)*

In addition to being a dilemma, finding a balance between abstinence and the acceptance of substance use also concerned disagreement within the team. For example, some team members regarded opioid maintenance treatment as substance use, while others saw it as a support to improve quality of life. The team seemed to have moved from seeing it as their main task to assist people regardless of their substance use, to focusing mainly on addressing substance use with the goal of total abstinence.

*“We need to ask what people are motivated for, which is not necessarily a change in substance use, and then focus on that. Because we work with change, and it doesn’t need to be about substance use.” (Interview 1)*

*“I think we agree that abstinence is the goal. I mean, that’s when people are free to live their life to the full? But to get there, you may need to believe that kind of life is worth living.” (Interview 3)*

After extensive discussions, the team had decided to adopt a 12-step approach at the time of the third interview. This involved an attitude that everyone can and should obtain total abstinence, and that addiction was the root of other problems. Within this approach, telling people to stop using alcohol or drugs and go to AA or NA meetings was seen as recovery-oriented practice.

*“For example a woman I’m working with who has severe alcohol problems… I’ve spent a lot of time telling her that she’s got to stop drinking completely. And I’ve recommended her to go to AA meetings. And then I’ve talked to her between those meetings, about how she felt about them. That’s a specific example of how I do recovery*-*oriented practice.” (Interview 3)*

Team members stressed that no one was rejected if they did not want total abstinence, but substance use was generally to be addressed first, and was seen as primary to mental health problems. Working towards total abstinence was experienced as difficult and ambitious, but also directing and inspiring.

*“We help everyone regardless of what they want, or we try to help them as best we can. But I think agreeing on what could be a good recovery process, I mean total abstinence, I think that helps us as much as it helps the people with the problems. So that we don’t get burned out, and (…) can even spread hope that it’s possible to get into a recovery process and have a better life, even if the problem is drugs.” (Interview 3)*

However, concern was raised that this approach would prevent individualised support and exclude people for whom abstinence was unrealistic, but who would still benefit from other services.

*“I feel we’re a local authority, we’re not a narrow niche, so I think we should include everyone. We need to face the facts, we have some substance users who may never stop, and we have a responsibility towards them.” (Interview 3)*

One way of addressing this dilemma was to balance the focus on total abstinence with other issues and work with social services to ensure basic needs.

*“When we get into chaotic situations, where everything’s a mess and so on, it’s important to sit down with the service user and put it down on paper, make priorities, and just clear away all that noise before you can focus properly and move on. If you don’t, those other things steal so much time and effort, so you have no chance (to work on the substance use problem).” (Interview 3)*

The team described that supporting people after they manage to stop taking alcohol or drugs was a priority, since this is a time when many people deal with issues such as loneliness, stressful life events, and housing or economic problems.

*“Then you’re off drugs, and you have no friends, your housing is bad, you have practically no activity, you start feeling a lot of emotions, everything you’ve been through. And we need to address that, and it demands a lot of us. It’s not like we tell people that once you quit drugs, everything will be fine. That’s when an even bigger job starts. It’s hard work for us, but it’s hardest for the person who takes the step and makes a change.” (Interview 3)*

## Discussion

Practitioners in a community mental health and addictions team experienced dilemmas related to recovery-oriented practice to support people with co-occurring disorders. These were balancing mastery and helplessness, balancing directiveness and a non-judgemental attitude, and balancing total abstinence and the acceptance of substance use.

Practitioners in the same team held different opinions on what recovery-oriented practice meant, and this was particularly apparent in addressing substance use. While recovery within mental health has increasingly been defined as possible regardless of symptom reduction, recovery in substance use has typically focused on abstinence [19]. While the concept of recovery *from* mental health problems is traditionally associated with a biomedical psychiatric approach [30], the concept of recovery as total abstinence from addictive stimuli is rooted in certain service user movements. The debate on abstinence versus harm reduction has generated large controversy in the substance use field during the past decade at least [31], with service users, policy makers, and practitioners on both sides of the debate, which extends to legalisation as well as medications in substance use treatment. While service users may disagree with the way practitioners define recovery in addiction [32], disagreement does not necessarily mirror the discourse of the recovery movement in mental health. The dilemma of abstinence versus accepting substance use described in this study seems partly related to this debate, and some team members communicated strong opinions on the fundamentality of abstinence. Further, it seems to relate to the complexity of addiction and to what may be a paradox rather than a dilemma: that harm reduction *and* abstinence may both be necessary approaches when addressing substance use in a recovery-oriented way.

While studies on first-person experiences of recovery in mental health have stressed the right to live well “within or despite symptoms” [30], first-person perspectives on substance use problems tend to stress at least some sort of control over substance use in order to enable recovery [3, 33]. This also appears in first-person experiences of co-occurring disorders [21, 34–36]. Importantly, there seems to be considerable individual variation in how substance use relates to recovery among people with co-occurring disorders [37], as well as in reasons for quitting substance use [38], and a categorical total abstinence approach seems to be at odds with recovery principles of supporting each individual’s goals and interests. Further, as is reflected in team members’ descriptions in the present study, demanding total abstinence in community services may indirectly exclude citizens from services. To people with co-occurring disorders, who already face the problem of falling between two stools in the health and social care system, this may mean a further alienation from fair access to services.

A central aspect of recovery-oriented practice is to empower people by supporting their own efforts in the recovery process. This implies sharing both power and responsibility. Life challenges, including structural factors, made the principle of empowerment problematic to practitioners in this study. Their descriptions of discrimination and unequal access to welfare goods resonate with critical voices that argue that focusing on empowerment without recognising structural factors may be destructive [12]. Balancing empowerment with fighting against, and compensating for, structural injustice seems highly important in recovery-oriented practice with this group of citizens. Interestingly, the team described moving towards sharing more responsibility for life changes by the time of the last interview. For example, they spent less time reaching out to those who did not attend services. This is not in accordance with guidelines, which recommend outreach services to people with co-occurring disorders. The terms noncompliance, nonadherence and dropout have been suggested as outmoded within a recovery-oriented system [39]. Also, patients who compliantly attend community services do not necessarily experience these services as helpful [40]. The issue of prioritising those who attend services may be seen as an example of competing priorities between recovery principles and structural demands, and illustrates that recovery-orientation depends on structural issues as well as training of staff [20].

Shared decision making about treatment goals was described as problematic because clients may have too low aspirations for change, hence needing directiveness. This is in line with the argument that shared decision making in the field of mental health is made difficult because practitioners, often incorrectly, do not think that patients know their own best [41]. This may be a universal phenomenon, indicating that such attitudes will need to be understood and addressed in order to achieve genuine shared decision making in the mental health and addictions field. When service users are perceived as unable to make decisions about their own life, directiveness will be a likely response from practitioners. Previous studies suggest that the usefulness and necessity of directiveness may be perceived differently by practitioners and service users. Coercion and paternalism have been seen as incompatible with recovery-oriented practice in qualitative studies with a service user perspective [8, 34]. Yet studies of practitioners’ accounts show that recovery-oriented practice and directiveness are not always seen as opposed to each other [14] and that authoritative behaviour by community mental health professionals may negatively affect therapeutic interactions, even when the professionals adopt a person-centred, recovery-oriented approach to practice [15]. An exploratory study of different levels of directiveness used by social workers in home health care found that disagreement between clients and social workers increased the risk of paternalistic action [42]. This resonates with descriptions in the present study that differing opinions of what a good life means may make it difficult to base treatment plans on the service user’s goals.

### Limitations and strengths

This article provides insights into practitioners’ experiences with dilemmas that may arise in recovery-oriented practice in the field of co-occurring disorders, a phenomenon which to our knowledge has not been explored in the research literature before. The methods used in this study do not allow for an immediate generalisation of the results, but the insights may have relevance to other contexts, and may direct future research. The results are based on participants’ descriptions of dilemmas in recovery-oriented practice as they appeared in group interviews. Other methods, such as participant observation or individual interviews, would have provided different descriptions. A case study approach would have enabled an exploration of the process of developing recovery-oriented practice in this particular context. However, the fact that interviews were carried out over 2 years enables insight into the changes over time. The service user perspective is not included directly in the data. Results are discussed with reference to studies of first-person experiences in order to counterbalance this limitation.

## Conclusion

Practitioners in a municipal mental health and addictions team presented several dilemmas related to recovery-oriented practice to support people with co-occurring disorders. Team members held different opinions on what recovery-oriented practice meant, particularly regarding how to address substance use. There is a need for further definition of recovery-oriented practice from different stakeholders’ perspectives. Innovative approaches to practice development and research that address the inherent dilemmas in recovery-oriented practice aimed at people with co-occurring disorders are needed.

## Additional files

**Additional file 1.** Interview schedule, first interview.

**Additional file 2.** Interview schedule, second and third interviews.
