# Supplementary material for: Dilemmas in recovery-oriented practice to support people with co-occurring mental health and substance use disorders: a qualitative study of staff experiences in Norway
Source: Int J Ment Health Syst. 2018 Jun 7;12:30. doi: 10.1186/s13033-018-0211-5 (PMC5992690; doi:10.1186/s13033-018-0211-5)
Supplement: Supplementary file 2 — Additional file 2. Interview schedule, second and third interviews. [file 13033_2018_211_MOESM2_ESM.docx]

**INTERVIEW SCHEDULE, SECOND AND THIRD INTERVIEW**

(Translated from Norwegian)

1: Thank you for accepting our request to interview you. We have reserved one hour, and there will be no breaks. Before we begin, I ask you to sign a consent form which is on the table in front of you. Participation is voluntary and you are free to withdraw your consent at any time. I will tape record the interview. The recording will be saved in a safe place and will be deleted when the project is over. The researchers are committed to confidentiality. It will not be possible to identify you in the published reports.

2: Thank you for participating in this interview. Your experiences may give valuable knowledge about what may lead to recovery in co-occurring disorders.

Today, I would like to know more about how you work, as of today, to support people with co-occurring disorders. I would like to know how you work, as well as your thoughts and experiences surrounding this.

We are interested in different opinions. Consensus is not a goal, rather the opposite! It is natural that you may hold different opinions, and I would like to hear those. There are no correct or wrong answers, and each one of you have valuable experience.

Sometimes people change their opinion during the interview, or think of new things, and that is also OK.

We would like to hear about what works well, and things that do not work so well, or where you may lack a good solution. It is your experience as practitioners that I would like to learn more about.

I will lead the interview and pose questions. I have some open questions and some detailed. It is my job to stick to the theme, so I might interrupt you at times. I will also summarise in order to check if I have understood what you said.

(Co-researcher) will observe and take notes, and she will help me to stick with the issue.

Are there any questions before we begin? Is it OK?

3: I would like to start with everyone saying their first name, profession, and how long you have been in the team.

4: What does recovery-oriented practice mean in this team? How do you work in a recovery-oriented way to support people with co-occurring disorders?

Can you please describe a course of intervention with a service user.

Can you please describe a normal day where you work to support people with co-occurring disorders?

Follow-up questions:

- Tell me more about that!

- I would really like to hear more about that.

- How do you work with that?

- What do you do to achieve that?

- Which experiences do you have with that?

- In which ways have that been useful?

- How does that feel like to you?

- What do the rest of you think about this?

7: Ending the interview

Is there anything that has not been said, that you would like to say?

Thank you for participating in this interview. You have contributed with valuable knowledge. If any of you should have questions afterwards, please contact me. My phone number is on the information sheet.

Thank you!
